# Neonatal fatal haemorrhage after a ritual circumcision: forensic and ethical considerations

**DOI:** 10.1007/s12024-025-01011-w

**Published:** 2025-04-24

**Authors:** Pierluigi Passalacqua, Raimondo Vella, Giorgio M. Coppola, Nazaria Lanzillo, Francesca Servadei, Michele Treglia, Margherita Pallocci

**Affiliations:** 1 Department of Public Health and Infectious Diseases, “Sapienza” University of Rome, Piazzale Aldo Moro, 5, Rome, 00185 Italy; 2 Department of Biomedicine and Prevention, University of Rome “Tor Vergata”, Via Montpellier, 1, Rome, 00133 Italy; 3 PhD Program in Applied Medical-Surgical Sciences, Department of Surgical Sciences, University of Rome “Tor Vergata”, Via Montpellier, 1, Rome, 00133 Italy

**Keywords:** Male circumcision, Hemorrhage, Fatal outcome, Forensic pathology, Ethics, Case reports

## Abstract

Neonatal circumcision is a common procedure worldwide, which may be performed for medical reasons and for cultural and religious motivations. Regarding ritual circumcision, there has been a wide-ranging debate in medical society about the level of acceptability of this practice. Even from a bioethical and legal point of view, the problem is approached differently in different contexts worldwide, especially given that, even if rare, complications can occur both during and after the procedure, and may result in infections, bleedings, hemorrhages and even death. Bleeding occurs most frequently after the fourth week of life and is related to the presence of an abundant venous vascularization of the penile shaft. Unlike adults, the blood loss rates suggestive for hemorrhagic shock are not defined in neonatal populations. Therefore, the diagnosis of the cause of death can be challenging for the forensic pathologist, especially if circumstantial information is missing. We report the case of a full-term infant boy born after a terminally complicated pregnancy. He underwent a “domestic” circumcision on 22nd day of life. The same day he was admitted to the emergency room in cardiac arrest and died despite resuscitation procedures. The autopsy findings revealed the presence of a large amount of blood in the diaper and a circumferential laceration of the penile shaft, consistent with a recent circumcision. Diffuse organ pallor was macroscopically and microscopically demonstrated, consistently with a hemorrhagic shock. To conclude, the diagnosis of hemorrhagic shock can be difficult in the newborn and requires the estimation of lost blood volume. Moreover, due to the possibility of rare fatal complications, neonatal circumcision should be performed only in a controlled, medical environment.

## Introduction

“Circumcision”, is a very ancient practice that has been developed independently and simultaneously in different cultures. Evidence of this procedure has been found, e.g. in the earliest Egyptian mummies (around 2300 BCE), and it is also attested by wall paintings that it was customary several thousand years earlier [1].

Nowadays, Male Circumcision (MC) is one of the most frequently performed operations worldwide, with a prevalence of 25–30% of the global male population [2], with higher rates in distinct geographical areas, such as North America, Australia and Africa [3, 4].

The reasons of MC are usually related to therapeutical, prophylactic or ritual purposes. Among the therapeutical indications, it is usually performed to treat preputial diseases, such as pathological phimosis or balanoposthitis [5].

Some authors have suggested that by improving penile hygiene, circumcision could represent a potential protective factor against infections of the urinary tract, sexually transmitted diseases, and inflammatory penile diseases [4].

In several cases, circumcision is also performed for religious and ritual aims, especially in newborns. Neonatal Male Circumcision (NMC) is required by several religions, such as Judaism– according to which it’s performed on 8th day of life -, Islam, as well as in several cultures of Africa and Australia (and particularly in Aboriginal population).

In such cases, NMC is not always performed by qualified and trained medical personnel, but also by relatives or representatives of their religious communities.

In this regard, it is important to notice that, though NMC is a substantially safe procedure, it can lead to potentially hazardous and rarely fatal complications, that can occur either during or after the procedure, and may result in infections, bleedings, hemorrhages and even death or SIDS [6–8].

A recent literary review has highlighted that the most frequent complications of therapeutical circumcisions involve adhesions, meatal stenosis and infections, while in the case of non-therapeutical ones, bleeding, infections and problems related to the removed of the device are observed [9]. Age is a determinant risk factor for complications, with a proportional increase in frequence, also in pediatric population. Another condition that may have an impact is the presence of pre-existing penile pathologies: indeed, the risk of complications is higher in case of therapeutic circumcisions, as well as in the case of operations performed by operators with a low level of experience and training and working in non-sterile environments [10].

The most serious complications, including death, are rare events, although a reliable estimate of the true incidence at global level is not currently available: according to some authors, complication rates may range from 0.2 to 5% up to 55% [11].

A retrospective analysis has estimated a case fatality rate of 10.2 deaths per 500,000 circumcisions. The same study showed that the concomitant presence of cardiovascular diseases or coagulopathies represents an increased risk of occurrence of fatal events in infants undergoing circumcision [12]. According to other authors, the incidence of fatal events is approximately 0.0012%, in most cases related to massive bleeding or infections [13].

Bleeding occurs most frequently after the fourth week of life and is related to the presence of an abundant both arterial and venous vascularization of the penile shaft [14].

The case we present concerns the death of a 22-days-old infant which, based on the circumstantial and clinical data, as well as of the findings of the judicial autopsy, could be causally linked to the procedure the infant had undergone a few hours before his death.

To the best of the authors’ knowledge, despite the extreme frequency NMC worldwide, the presented case is among the few described concerning the fatal consequences of hemorrhagic shock in an infant undergoing such a procedure.

## Case description

We report the case of a full-term infant boy born via an urgent caesarean section after a terminally complicated pregnancy. The delivery occurred at 40 weeks + 4 and the C-section was due to a persistent bradycardia at the CTG. APGAR Score was 9/10 and the infant showed normal parameters of development (weight 3020 g; length 48 cm, cranial circumference 35 cm). No congenital abnormalities, except for the presence of a sebaceous cyst on the prepuce, were evident and he was discharged after 5 days of clinical observation. From the evidence of the investigation, the infant underwent a “domestic” circumcision on 22nd day of life. After a few hours, he was admitted to the emergency department due to cardiac arrest. He presented suggestive signs for hemorrhagic shock (pale and cold skin), state of unconsciousness (Glasgow Coma Scale 3) and bleeding from a circumferential wound of the penile shaft. He died 25 min after admission, despite continuous resuscitation procedures.

Therefore, the Public Prosecutor demanded for a forensic investigation and ordered a forensic autopsy to be performed.

**Fig. 1:**
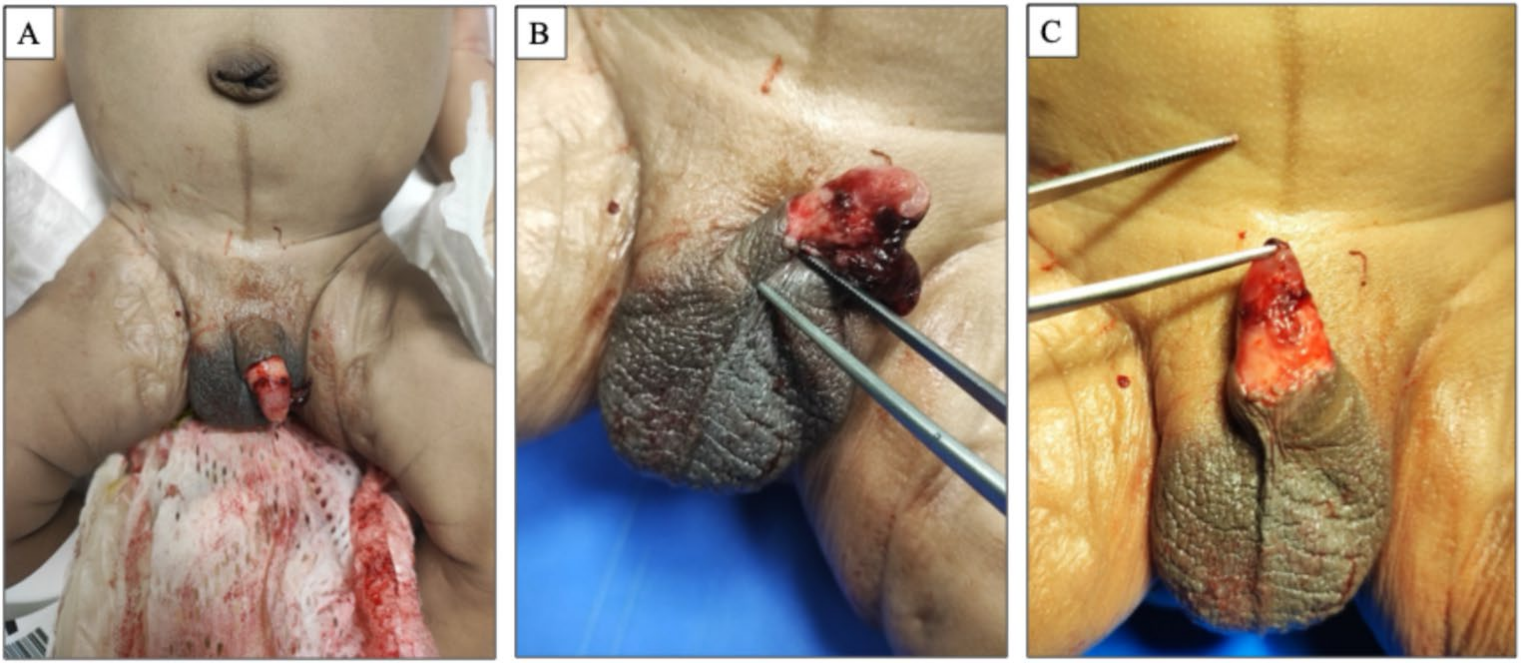
**a**: significant amounts of blood in the diaper; **b**: voluminous clot at the penile shaft and skin lesion; **c**: after removing the clot, the skin lesion is revealed, which at the level of the ventral surface of the penile shaft is characterised by ragged edges

The autopsy was performed 3 days after death. The total body weight was 3530 g. All the routinary anthropometric measurements were taken: cranial circumference was 35 cm; chest circumference was 35 cm; abdominal circumference was 36 cm; body length was 54 cm; crown-rump length was 37 cm; upper limbs length was 22 cm; lower limbs length was 21 cm; the foot length was 8.5 cm. The external examination of the corpse revealed diffuse skin and mucosal pallor. In the diaper, the presence of a huge blood amount with urine and liquid feces was evident. A blood clot, which was removed and sampled for histological analysis, was attached to the mucous tissue of the left lateral portion of the penile shaft. The penis was 3 cm in length and showed the presence of a circumferential laceration of the shaft located at 1.8 cm from the base, characterized by irregular shape and involving the subcutaneous tissues, consistently with a recent circumcision (Fig. 1a, b and c).

No external signs suggestive for violence were found.

**Fig. 2:**
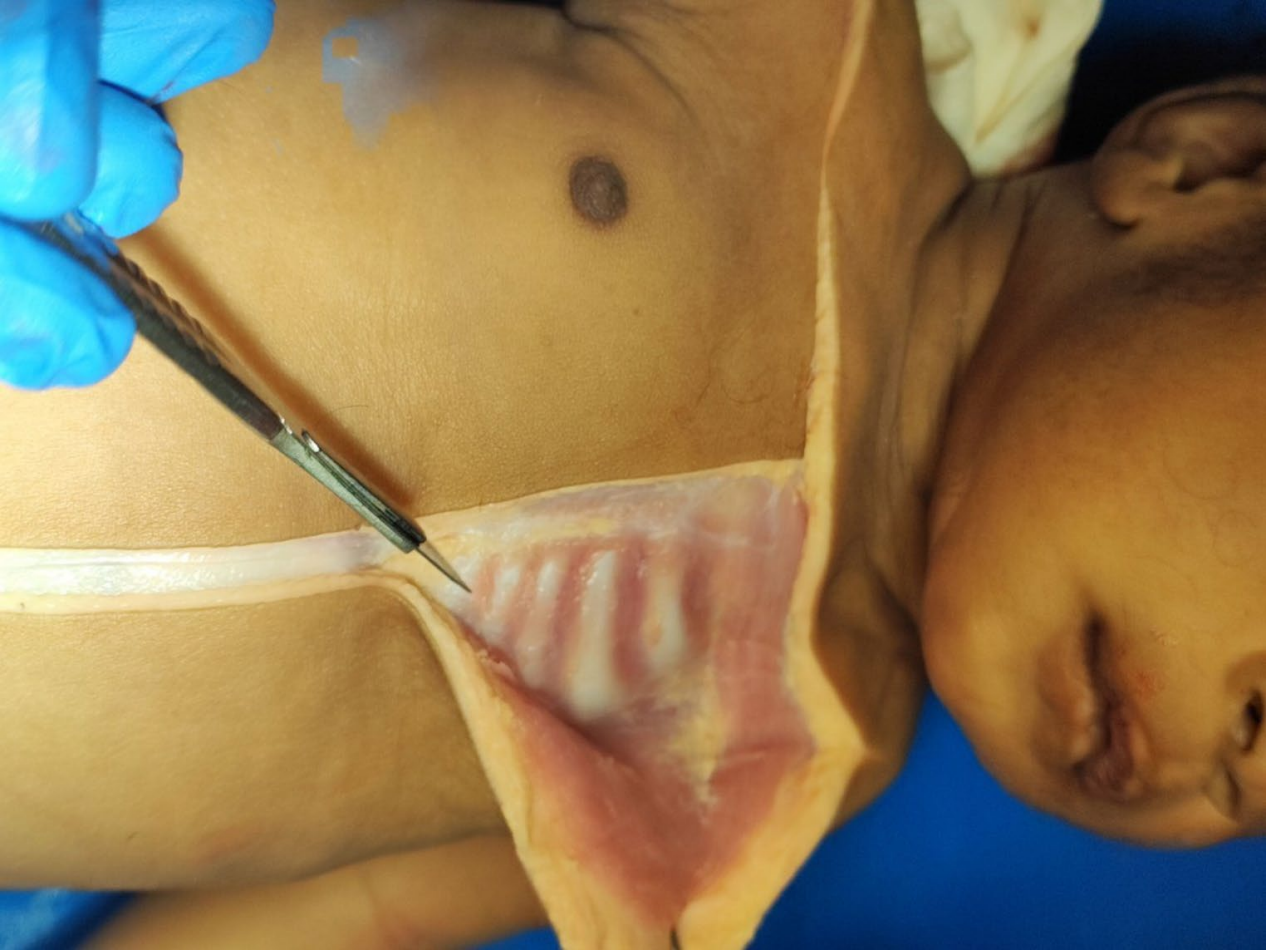
Intense pallor of the intercostal muscles

Gross examination of the internal organs did not revealed abnormalities, except for an intense pallor of the organs and the muscular structures (Fig. 2).

The section of the head showed the absence of traumatic alterations. Brain’s weight was 550 g, and no parenchymal alterations were macroscopically detected. The rib cage was integrous. The inspection of the thoracic structures revealed a diffuse pallor in the musculoskeletal structures, as per hypoxaemia. Kidneys presented smooth surface and were characterized by cortical pallor and slightly hyperemic papillae, with hyperemic rim at cortico-medullary border.

Anatomical dissection of the penis did not reveal any structural abnormalities.

Organ samples were taken and prepared for microscopical analysis and stained with hematoxylin-eosin.

**Fig. 3:**
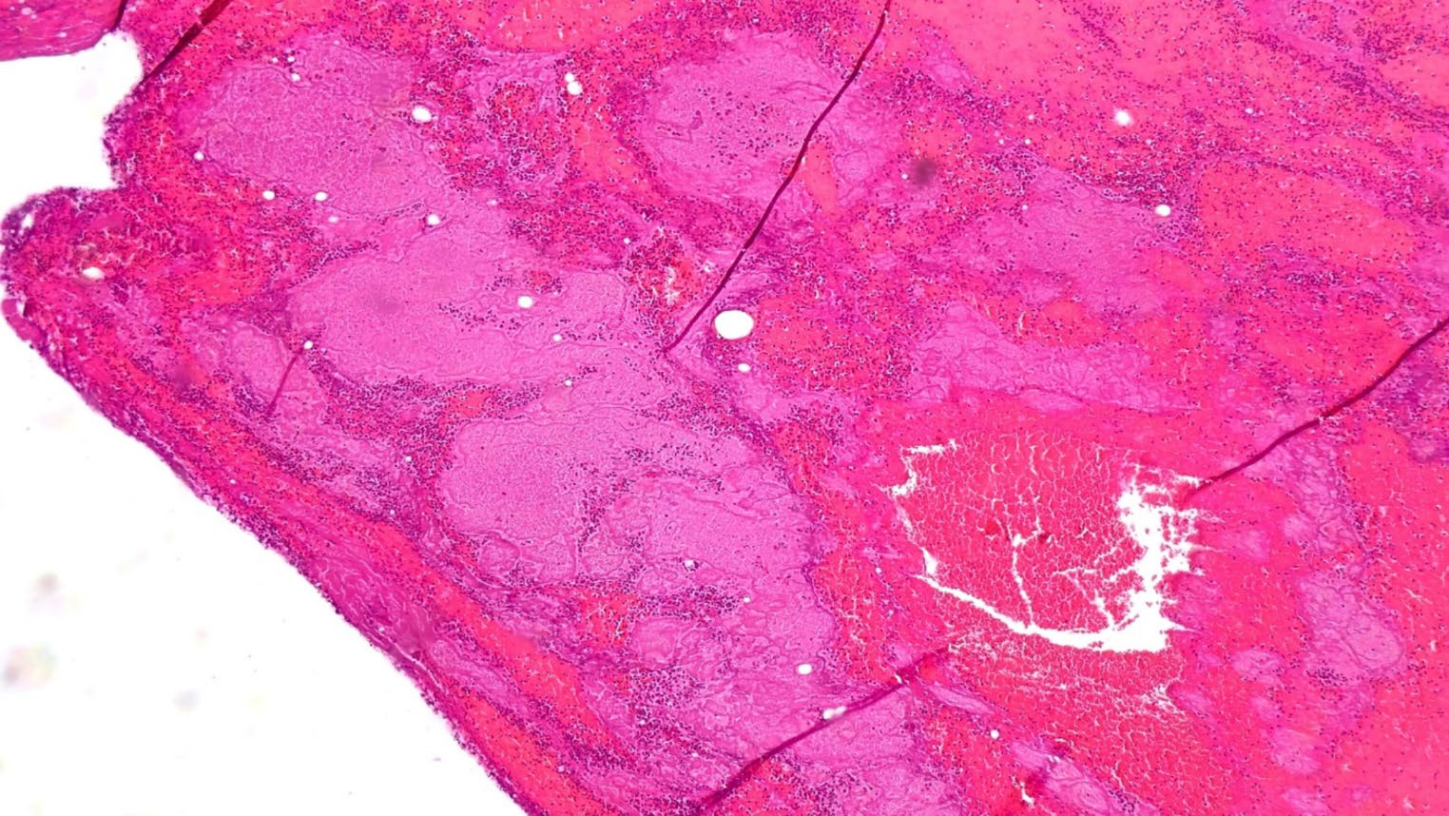
Histologic appearance of the of the blood clot adhered to the penile wound characterised by the presence of fibrin and blood cells

The specimens taken from the clot showed the presence of hematic and fibrinous material encompassing inflammatory cells, these predominantly represented by neutrophil granulocytes (Fig. 3).

Consistently, the specimens taken from the penile wound also showed vascular congestion and presence of haematous fibrin material with neutrophil granulocytes. The microscopical observation of the histological specimens revealed the presence of hypoxia and ischaemia in the encephalon, heart, spleen, kidneys, thymus and adrenal glands.

The integrated analysis of the autoptic and histological investigations, as well as the circumstantial data, could lead to the identification the cause of death as a hemorrhagic shock due to the injury to the penis occurred during the circumcision.

## Discussion

In the presented case, the autoptic and histological findings where suggestive for a hemorrhagic shock occurred after a “domestic” NMC.

The aim of circumcision is the removal of the preputial skin in order to keep the glans uncovered. Even though NMC is a daily performed procedure all over the world, from a forensic point of view the fatal cases resulting from postoperative complications are rarely described in literature and are generally related to procedures performed by inexperienced or non-medical personnel [10, 13, 15, 16].

The case described by Hiss et al. [15] also concerned a newborn who, after a ‘ritual’ circumcision performed in a domestic environment, developed a severe and ultimately fatal hemorrhagic shock. In that case, however, the histological investigations revealed a hepatopathy that contributed to the determination of the hemorrhagic shock.

Cohen et al. [17], reported a case of hemorrhagic shock after circumcision given to a severe congenital Factor XIII deficiency.

In our case, an analysis of the available data, did not reveal any previous condition that could have influenced the fatal outcome, which was therefore entirely attributable to the ‘’surgical’’ procedure and the conspicuous blood loss that followed.

The fundamental forensic aspect of the presented case to be considered is the reconstruction of the pathophysiological cascade that led to death.

Unlike adults, in neonatal population, the blood loss rates suggestive for hemorrhagic shock are not defined and they are usually expressed, as suggested by some authors, as a percentage of total circulating volume: a blood loss below 15% of the total volume usually demonstrates only mild symptoms such as tachycardia or anxiety (Class I); by a loss between 15 up to 25%, oliguria, mild tachypnea, irritability, and mottled cool extremities can be observed, as early signs of diminished end organ perfusion (Class II); in Class III, corresponding to a loss between 26 and 39%, patients show significant tachycardia, hypotension, irritability (and even lethargy), pale extremities and develop metabolic acidosis; if the loss is greater than 40% total blood volume (Class IV), they become lethargic or may lose consciousness and show a severe tachypnea and tachycardia, significant hypotension and acidosis, as well as cyanosis and anuria, as a result of the mismatch.

Regarding our case, since the circulating blood volume cannot be precisely quantified, it can be calculated according to scientifically accepted estimates and formulas, based on weight and height [18].

In the present case, the circulating blood volume could be estimated within a range of approximately 230.75–356.46 ml. Consequently, it was therefore possible to consider that the onset of a severe hemorrhagic shock beyond stage IV of the above-mentioned classification, could be the consequence of the rapid blood loss of at least 92.3 to 142.59 ml.

It should be however underlined that these values are only estimates, because in the case presented it was not possible, based on the evidence available, to estimate more accurately the extent of the blood loss suffered by the newborn or the precise timing of the onset of bleeding.

In accordance with the literature, the incidence of bleeding during circumcision varies from 0.1% up to 35% [11] and this appeared to be significantly higher if the NMC is performed after the 4th day of life [19].

In this frame, Edler et al. [2] have reported several cases of complications, including 4 cases of hemorrhagic shock (one of whom resulting in death) and argued that given the frequence of potentially fatal complications, NMC should be performed only by medical trained professionals and in hospitals with 24-h emergency departments.

Mano et al. described the characteristics and outcomes of post-ritual circumcision bleeding complications, as well as the short-term outcome [20], suggesting the indication for admission in circumcised children presenting active bleeding at the ER.

Another factor to be considered in the determinism of death in our case is the possible delay in transferring the infant to hospital which may also have played a role.

Given the possibility of potentially fatal complications, a number of authors, as well as jurists and bioethicists, have argued that performing circumcision in healthy individuals without medical indications raises an ethical and medico-legal issue, since it must be considered that in such cases it is a surgical procedure performed on a structurally healthy individual without any health or preventive indication and practiced on minors, thus without the consent of the person concerned [4, 16, 21–23].

In this frame, medical and academic associations have no uniform agreement, and the preventive effectiveness of its execution is still debated and controversial [24].

In the U.S., Medicaid (the State-level Insurance) has defunded the coverage of NMC after the AAP indicated that the procedure, due to a lack of evidence, was not essential to the child’s well-being and that the decision was best left to parents but resulting to a decrease in frequency of the number of prophylactic circumcisions [25, 26].

Later in 2012, the American Academy of Pediatrics (AAP) stated that the benefits, in terms of prevention, of routinary circumcision outweighed the risks [27].

On the other hand, the British Medical Association (BMA) asserts that circumcision for purely prophylactic or ritual purposes is not automatically justified by parental consent and urges doctors to inform parents of the issues involved in an invasive medical operation [16].

This controverted efficacy and legitimacy of the procedure therefore led some Countries (e.g. Norway) [23] to consent health professionals the possibility to express conscientious objection to the procedure.

Indeed, the legislation of many countries, moreover, prohibits body modification even in the presence of valid informed consent.

In 2007 an Italian Court [16], emphasized that thought male circumcision constitutes a violation of the psychophysical integrity of a subject who is generally unable to express his consent effectively, it has long been amply accepted by Western custom and culture. However, the same Court also ruled that NMC was a medical act that necessarily had to be performed by medical personnel, according to the Italian Law and that it must be performed in accordance with good clinical practice.

Later, in 2012 a sentence of a German Court, by considering the legal aspects of ritual circumcision, ruled that non-therapeutic circumcision constitutes a bodily assault that violates the child’s right to autonomy and self-determination and that the procedure should be delayed until an age at which the boy can consent on his own [28].

Conclusively, in view of the possibility of complications and fatal outcome, a neonatal circumcision, whatever its reason, takes on the character of medical procedure and should be performed only in medical and safe environment.

Moreover, since this procedure is performed on subjects incapable of expressing valid consent, restricting theoretically their right to self-determination, further ethical and legal reflection about the evaluation of the lawfulness of this practice on minors should be consequently appropriate.

## Conclusions

In the case presented, the reconstruction of the causal chain of the procedure with the cause of death, as well as the determination of its biological plausibility was only possible after the estimation of the circulating blood volume and of the blood loss and by their association with the objective autoptic and histological findings.

Therefore, the diagnosis of hemorrhagic shock can be difficult in the newborn and requires the estimation of lost blood volume. Moreover, due to the possibility of rare fatal complications like hemorrhagic shock neonatal circumcision should be performed only in a controlled, medical environment.

Regarding the current regulatory system, the performance of such a procedure by non-medical personnel may integrate, in some Countries, the crimes of personal injury and abusive medical practice.

A particular ethical and legal reflection about the evaluation of the lawfulness of this practice on minors should be appropriate, especially in case of absence of clinical indication, due to the lack of valid informed consent.

## Key points

1. The case concerns an infant who died following a ritual circumcision procedure.

2. Male circumcision is a procedure that can be associated with rare complications as far as death.

3. One of the major risks in the neonatal period is uncontrolled bleeding which, as in the present case, can lead to death because of hemorrhagic shock.

4. In the present case, the circulating blood volume could be estimated within a range of approximately 230.75–356.46 ml. Consequently, it was therefore possible to consider that the onset of a severe hemorrhagic shock could be the consequence of the rapid blood loss of at least 92.3 to 142.59 ml.

5. The ethical and legal aspects of neonatal circumcision essentially concern cases of circumcision practiced for non-health reasons (religious or cultural) and by individuals who do not have the appropriate professional qualifications, situations that are considered differently in different national and legal contexts.
